# EXAMINING THE NEEDS OF PEOPLE WITH DEMENTIA AND FAMILY CARERS DIRECTLY AFTER THE DIAGNOSIS

**DOI:** 10.1093/geroni/igz038.3259

**Published:** 2019-11-08

**Authors:** Claudia van der Velden, Iris van Asch, Elsemieke van belzen

**Affiliations:** Netherlands Institute of Mental Health and Addiction (Trimbos-institute), Department on Aging, Utrecht, Netherlands

## Abstract

Most often the diagnosis dementia gives people clarification, but at the same time it raises questions and causes a range of emotions. According to professionals, support for people with dementia and family carers (dyads) nowadays is mainly focused on later-stage-dementia instead on early-stage. Besides, this support is mostly about practical/medical topics and less about emotional/psychological or social topics. Studies show that it is a complex process dyads to cope with the diagnosis in their daily life directly after diagnosis. The aim of the present study was to examine the specific needs of dyads in order to support them in the early-stages after diagnosis. Focus groups, interviews and a literature-review were performed to determine these needs. Together with professionals, the findings were translated into a conversation guide, which helps professionals to have a structured dialogue on practical/medical, emotional/psychological and social topics to support dyads in an early-stage after diagnosis. An evaluation is conducted by observations and interviews with dyads and a work-session with professionals. People with dementia, their caregiver(s) and professionals experienced these conversations as very positive. Dyads indicated the type of counsellor (accessible, warm and open), timing of the conversation and topics about ‘not making the diagnosis too heavy’ and ‘sharing the diagnosis with others’ as important. The professionals were also very positive about the conversations. Important themes for them were focusing on the more personal, holistic and preventive approach in the conversations.

